# Dataset for understanding why people share their travel experiences on social media: Structural equation model analysis

**DOI:** 10.1016/j.dib.2020.105447

**Published:** 2020-03-19

**Authors:** Tiago Oliveira, Benedita Araujo, Carlos Tam

**Affiliations:** NOVA Information Management School (NOVA IMS), Universidade Nova de Lisboa, Campus de Campolide, 1070-312 Lisboa, Portugal

**Keywords:** Sharing travel experiences data, Portuguese tourism data, Social media, Actual travel experience sharing, Social influence theory

## Abstract

The data presented in this article relates to the individual intrinsic and extrinsic motivations to share travel experience in social media. The 381 records were gathered in Portugal using an online survey. A statistical analysis of the data was carried out using partial least squares (PLS). This dataset shows a relationship between identification, internalization, and compliance to perceived enjoyment, and also, between perceived enjoyment, altruistic motivations, personal fulfillment, and self-actualization as well as security and privacy reasons to actual travel experience sharing. For further findings and interpretation, please refer to the research article entitled “Why do people share their travel experiences on social media?” [1]. We suggest the use of this data to compare with data collected by other researchers to develop cross-country analyses based on the model proposed by Oliveira, Araujo, and Tam [1].

Specifications table

**Table:** 

Subject	Tourism, Leisure, and Hospitality Management
Specific subject area	Sharing tourism experiences through social media
Type of data	Table Figure
How data were acquired	Survey
Data format	Raw
Description of data collection	We gathered the data using an online survey through Google forms. We tested our framework by submitting a survey through Facebook between June 2017 and July 2017. The participants of this dataset are Portuguese persons who use Facebook.
Data source location	Portugal
Data accessibility	With the article
Related research article	Oliveira, T., Araujo, B., & Tam, C. (2020). Why do people share their travel experiences on social media? Tourism Management, 78 (2020), 104041

## Value of the data

• The dataset is useful because it can be used as a reference for understanding why people share their travel experiences on social media or not.
• This data is beneficial for all parties involved, especially for travel marketers and tourism agencies.
• This data can help to understand the drivers that lead people to participate online to tell their travel experiences to others, as well as to gain insight on the factors that lead them not to share.
• Finally, this data can be used for researchers to develop a cross-country comparison model, i.e., comparing their findings with the model published in “Oliveira, T., Araújo, B., & Tam, C. (2020). Why do people share their travel experiences on social media? Tourism Management, 78 (2020), 104041″.

## 1 Data

The data file spreadsheet accompanying this article consists of 381 rows and 32 columns of data. Each row represents an individual's response to a questionnaire. A seven-point range scale was used to allow the respondents to indicate how much they agree or disagree with a particular statement, so a numerical value in the data file means the respondent level of agreement, with 1 being "strongly disagree" and 7 being "strongly agree". Our demographic data indicated that of the 381 respondents, 251 (66%) are female. Regarding age, 120 (31%) of the respondents are under 24 years, 184 (48%) of the respondents are between 25 and 44 years, and the rest (77 respondents) are above 44 years. Regarding the highest level of education completed, the majority of respondents are undergraduate 208 (55%), followed by masters’ degree 121 (32%).

Each questionnaire item in the columns was given a label, as shown in the first row. Iden is the short form for identification; Inter for internalization; Comp for compliance; Pjoy for perceived enjoyment; AS for actual travel experience sharing; AM for altruistic motivations; PF for personal fulfilment and self-actualization; ER for environmental reasons; PR for personal reasons; RR for relationship reasons; and SR for security and privacy reasons. After filtering the data and the application of the measurement model, three items of identification remained for the structural equation modelling analysis: Iden1, Iden2 and Iden3; three items of internalization: Inter1, Inter2 and Inter3; three items of compliance: Comp1, Comp2 and Comp3; three items of Perceived enjoyment: Pjoy1, Pjoy2 and Pjoy3; three items of actual travel experience sharing: AS1, AS2 and AS3; three items of altruistic motivations: AM1, AM2 and AM3; three items of personal fulfilment and self-actualization: PF1, PF2 and PF3; two items of environmental reasons: ER2 and ER3; three items of personal reasons: PR1, PR3 and PR4; two items of relationship reasons: RR2 and RR3; and three items of security and privacy reasons: SR1, SR2 and SR3 (see Table 1 below).

**Table 1:** The data file items.

Constructs	Items remaining after measurement model
Identification	Iden1, Iden2, Iden3
Internalization	Inter1, Inter2, Inter3
Compliance	Comp1, Comp2, Comp3
Perceived enjoyment	Pjoy1, Pjoy2, Pjoy3
Actual travel experience sharing	AS1, AS2, AS3, AS4
Altruistic motivations	AM1, AM2, AM3
Personal fulfillment and self-actualization	PF1, PF2, PF3
Environmental reasons	ER2, ER3
Personal reasons	PR1, PR3, PR4
Relationship reasons	RR2, RR3
Security and privacy reasons	SR1, SR2, SR3

## 2 Experimental design, materials, and methods

We gathered the data using an online survey through Google forms. We tested our framework by submitting a survey through Facebook between June 2017 and July 2017. The participants of this dataset are Portuguese persons who use Facebook. These data were provided in a Microsoft Excel Worksheet as supplementary data for this article. Data were analysed applying statistical tests including the partial least squares structural equation model (PLS-SEM) approach. We used SmartPLS 3.0 software [2].

We assessed the composite reliability criterion to verify the internal consistency. The values in Table 2 showed scores greater than 0.7 [3,4]. The average variance extracted (AVE) was evaluated based on Table 2; all items presented values above 0.5 [5]. Discriminate validity was validated based on three criteria: Fornell–Larcker criteria (please, see Table 3) [6], cross-loading [7], and heterotrait–monotrait ratio of correlations (HTMT) [8]. All criteria reveal that the measurement model presents discriminant validity (please, see in [1]).

**Table 2:** Construct reliability and average variance extracted.

Constructs	Composite Reliability	Average variance extracted (AVE)
Identification (Iden)	0.931	0.819
Internalization (Inter)	0.894	0.739
Compliance (Comp)	0.805	0.588
Perceived enjoyment (Pjoy)	0.971	0.918
Actual travel experience sharing (AS)	NA	NA
Altruistic motivations (AM)	0.937	0.831
Personal fulfilment and self-actualization (PF)	0.954	0.873
Environmental reasons (ER)	0.818	0.699
Personal reasons (PR)	0.831	0.624
Relationship reasons (RR)	0.900	0.818
Security and privacy reasons (SR)	0.943	0.847

**Table 3:** Fornell–Larcker Criterion (the square root of average variance extracted (AVE) shown in bold on the diagonal).

Factors	Iden	Inter	Comp	Pjoy	AS	AM	PF	ER	PR	RR	SR
**Iden**	**0.905**										
**Inter**	0.679	**0.859**									
**Comp**	0.383	0.363	**0.767**								
**Pjoy**	0.597	0.643	0.190	**0.958**							
**AS**	0.538	0.562	0.144	0.747	NA						
**AM**	0.376	0.377	0.271	0.443	0.503	**0.912**					
**PF**	0.671	0.623	0.367	0.571	0.564	0.313	**0.934**				
**ER**	−0.107	−0.148	0.144	−0.347	−0.250	−0.168	−0.103	**0.836**			
**PR**	−0.220	−0.263	0.093	−0.455	−0.422	−0.184	−0.191	0.439	**0.790**		
**RR**	−0.303	−0.319	−0.018	−0.513	−0.470	−0.264	−0.305	0.433	0.687	**0.904**	
**SR**	−0.212	−0.255	0.009	−0.420	−0.425	−0.121	−0.265	0.308	0.611	0.592	**0.920**

For formative construct (actual travel experience sharing (AS)) we based on Table 4. We can see that problems in terms of multicollinearity are not present because the variance inflation factor (VIF) is lower than the value of 5 [9]. Based on Table 4 all items are statistically significant; this element reveals the adequacy of the items that belong to this formative construct.

**Table 4:** Measurement model of formative construct.

Formative construct	Items	VIF	Weights	T statistics
actual travel experience sharing (AS**)**	AS1	1.529	0.763	12.954
	AS2	1.662	0.169	2.809
	AS3	1.532	0.157	2.816
	AS4	1.398	0.175	2.498

**Fig. 1:**
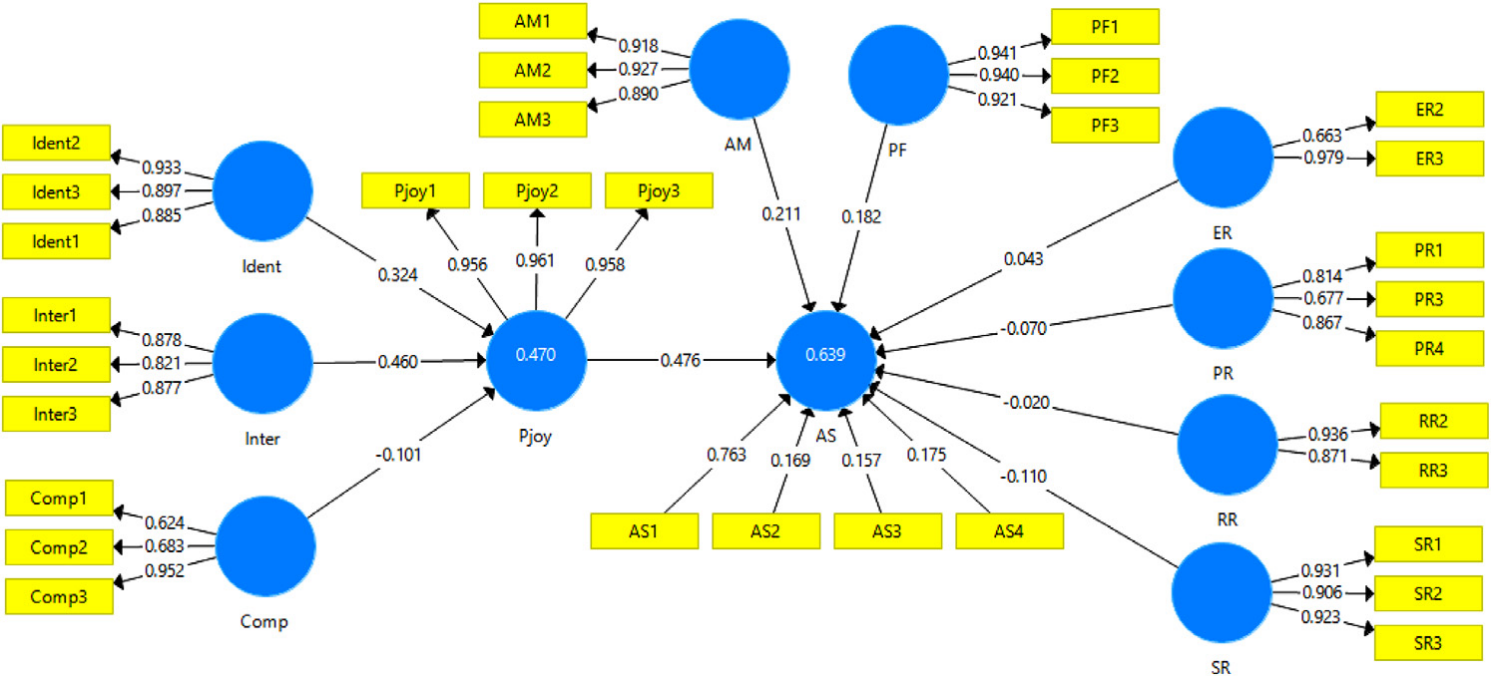
Pictorial of the research model.

Table 5 summarizes the path coefficients of the variables showing ten paths, seven paths are supported, and three are not supported. The path coefficients and r-squares of this model are in Fig. 1.

**Table 5:** Path coefficient of the variables.

Path	Path coefficients	T statistics	*P*-values
Ident -> Pjoy	0.324	5.891	0.000
Inter -> Pjoy	0.460	8.223	0.000
Comp -> Pjoy	−0.101	2.057	0.040
Pjoy -> AS	0.476	7.479	0.000
AM -> AS	0.211	3.384	0.001
PF -> AS	0.182	3.997	0.000
ER -> AS	0.043	1.163	0.245
PR -> AS	−0.070	1.318	0.187
RR -> AS	−0.020	0.380	0.704
SR -> AS	−0.110	2.332	0.020

Note: PLS estimation (*p < 0.05; **p < 0.001).

## Declaration of interests

The authors declare that they have no known competing financial interests or personal relationshipsthat could have appeared to influence the work reported in this paper.

## Appendix Supplementary materials

Image, application 1

Image, application 2
