# Supplementary material for: Dataset for understanding why people share their travel experiences on social media: Structural equation model analysis
Source: Data Brief. 2020 Mar 19;30:105447. doi: 10.1016/j.dib.2020.105447 (PMC7168349; doi:10.1016/j.dib.2020.105447)
Supplement: Supplementary file 2 [file mmc2.docx]

**Constructs and items**

| **Construct** | **Items** | **Description** | **Reference** |
| --- | --- | --- | --- |
| Identification | Iden1 | I am very interested in what group members think about travel experience sharing. | [1] |
|  | Iden2 | I feel a sense of belonging to a group when I share my travel experiences through social media. |  |
|  | Iden3 | I feel I will fit into a group when I share my travel experiences through social media. |  |
| Internalization | Inter1 | The reason I prefer to share my travel experiences is primarily based on the similarity of my values and those represented by social media. | [1] |
|  | Inter2 | The reason I prefer to share my travel experiences on social media than on other communication tools is because of its value. |  |
|  | Inter3 | I want to share my travel experiences on social media because I think it is congruent with my values and beliefs. |  |
| Compliance | Comp1 | Unless I am rewarded for sharing my travel experiences on social media in some way, I may spend less time sharing knowledge and information. | [1] |
|  | Comp2 | How hard I work on sharing my travel experiences is directly related to how much I am rewarded. |  |
|  | Comp3 | In order for me to get the responses I want on social media it is necessary to express the right behaviour or attitude on social media. |  |
| Perceived Enjoyment | Pjoy1 | I usually find sharing my travel experiences through social media to be enjoyable. | [1] |
|  | Pjoy2 | Sharing my travel experiences through social media in a group is pleasant. |  |
|  | Pjoy3 | I have fun sharing my travel experiences through social media in a group. |  |
| Actual travel experience sharing (1) |  | Please choose your usage frequency for each of the following: | [2] |
|  |  | Note: Frequency ranged from 0 “never” to 7 “more than once per day of the trip” |  |
|  | AS1 | a) Every time I travel I share photos |  |
|  | AS2 | b) Every time I travel I share videos |  |
|  | AS3 | c) Every time I travel I share personal blogs |  |
|  | AS4 | d) Every time I travel I share reviews on TripAdvisor or other websites from hostels and restaurants I visited. |  |
| Altruistic Motivations | AM1 | I want to help others | [3] |
|  | AM2 | I want to prevent people from using bad products |  |
|  | AM3 | I want to contribute to websites that are useful for me |  |
| Personal fulfilment and self-actualization | PF1 | I want to be recognized because of my travel experiences | [3] |
|  | PF2 | I like to transmit what I want people to think of me |  |
|  | PF3 | It is important to me that people know I travel |  |
| Environmental reasons |  | Please choose the extent to which each of the hypotheses below inhibit online participation: |  |
|  | ER1 | There is a long delay in response to postings | [4] |
|  | ER2 | There is poor quality of messages |  |
|  | ER3 | The interaction design is bad |  |
|  | ER4 | There is a low response rate |  |
| Personal reasons | PR1 | I had no confidence in sharing my travel experiences on social media. | [4] |
|  | PR2 | I am afraid that what I post may not be important, may not be completely accurate, or may not be relevant to a specific discussion. |  |
|  | PR3 | I am too shy to share travel experiences in public. |  |
|  | PR4 | I do not post because my needs, such as searching for information, could be fully satisfied by lurking (reading is enough) |  |
| Relationship reasons | RR1 | I am afraid to make a commitment to a group. | [4] |
|  | RR2 | I do not want to spend additional time and resources to maintain a commitment. |  |
|  | RR3 | I have low intimacy with other members |  |
| Security and privacy reasons | SR1 | My requirements for security and privacy are not satisfied by sharing my travel experiences. | [4] |
|  | SR2 | I am afraid that sharing my travel experiences will place me in danger or reveal my personal information. |  |
|  | SR3 | One of the main reasons for not sharing my travel experiences is to preserve privacy and safety |  |

**References**

[1] M. Kang, M. a Schuett, Determinants of sharing travel experiences in social media, Journal of Travel & Tourism Marketing. 30 (2013) 93–107. doi:10.1080/10548408.2013.751237.

[2] V. Venkatesh, J. Thong, X. Xu, Consumer acceptance and user of information technology: Extending the unified theory of acceptance and use of technology, MIS Quarterly. 36 (2012) 157–178.

[3] A.M. Munar, J.K.S. Jacobsen, Motivations for sharing tourism experiences through social media, Tourism Management. 43 (2014) 46–54. doi:10.1016/j.tourman.2014.01.012.

[4] N. Sun, P.P.-L. Rau, L. Ma, Understanding lurkers in online communities: A literature review, Computers in Human Behavior. 38 (2014) 110–117. doi:10.1016/j.chb.2014.05.022.
